# The Cascade [1,5]-Hydride Shift/Intramolecular C(sp^3^)–H Activation: A Powerful Approach to the Construction of Spiro-Tetrahydroquinoline Skeleton

**DOI:** 10.3389/fchem.2022.840934

**Published:** 2022-04-07

**Authors:** Hongmei Liu, Yunyun Quan, Long Xie, Xiang Li, Xin Xie

**Affiliations:** ^1^ Department of Pharmacy, Personalized Drug Therapy Key Laboratory of Sichuan Province, Sichuan Provincial People’s Hospital, School of Medicine, University of Electronic Science and Technology of China, Chengdu, China; ^2^ Translational Chinese Medicine Key Laboratory of Sichuan Province, Sichuan Academy of Chinese Medicine Sciences, Sichuan Institute for Translational Chinese Medicine, Chengdu, China; ^3^ State Key Laboratory of Southwestern Chinese Medicine Resources, Hospital of Chengdu University of Traditional Chinese Medicine, School of Pharmacy and College of Medical Technology, Chengdu University of Traditional Chinese Medicine, Chengdu, China

**Keywords:** cascade reaction, [1.5]-hydrogen transfer, intramolecular C(sp^3^)-H activation, spiro-tetrahydroquinoline, *tert*-amino effect

## Abstract

The direct functionalization of inert C–H bonds is regarded as one of the most powerful strategies to form various chemical bonds and construct complex structures. Although significant advancements have been witnessed in the area of transition metal-catalyzed functionalization of inert C–H bonds, several challenges, such as the utilization and removal of expensive transition metal complexes, limited substrate scope and large-scale capacity, and poor atom economy in removing guiding groups coordinated to the transition metal, cannot fully fulfill the high standard of modern green chemistry nowadays. Over the past decades, due to its inherent advantage compared with a transition metal-catalyzed strategy, the hydride shift activation that applies “*tert*-amino effect” into the direct functionalization of the common and omnipresent C(sp^3^)–H bonds adjacent to *tert*-amines has attracted much attention from the chemists. In particular, the intramolecular [1,5]-hydride shift activation, as the most common hydride shift mode, enables the rapid and effective production of multifunctionally complex frameworks, especially the spiro-tetrahydroquinoline derivatives, which are widely found in biologically active natural products and pharmaceuticals. Although great accomplishments have been achieved in this promising field, rarely an updated review has systematically summarized these important progresses despite scattered reports documented in several reviews. Hence, in this review, we will summarize the significant advances in the cascade [1,5]-hydride shift/intramolecular C(sp^3^)-H functionalization from the perspective of “*tert*-amino effect” to build a spiro-tetrahydroquinoline skeleton, and the content is categorized by structure type of final spiro-tetrahydroquinoline products containing various pharmaceutical units. Besides, current limitations as well as future directions in this field are also pointed out. We hope our review could provide a quick look into and offer some inspiration for the research on hydride shift strategy in the future.

## Introduction

Undoubtedly, the functionalization of inert C–H bonds is one of the most effective and powerful tools for the formation of various chemical bonds in modern organic synthesis (Su et al., 2015; Qin et al., 2017; Karimov and Hartwig, 2018; Sauermann et al., 2018). Over the past decades, tremendous advancements have been witnessed in this dynamic field, especially in the direct functionalization of unreactive C–H bonds (Hartwig, 2012; Mousseau and Charette, 2013; Yang, 2015; Hartwig, 2016; He et al., 2019). Compared to classic transition metal-catalyzed coupling reactions, the direct modification of ubiquitous C–H bonds of simple organic compounds, without pre-activation and generation of a large number of wastes such as halides and tedious synthetic procedures, has attracted intense interest from the academic and industrial community (Peng and Maulide, 2013; Zheng and You, 2014; Qin et al., 2017; Sauermann et al., 2018). Owing to its intrinsic advantages, numerous innovative and efficient synthetic methodologies have been successfully explored, offering a straightforward access to rapidly synthesize structurally complex molecules (Hazelard et al., 2017; Karimov and Hartwig, 2018; Hong et al., 2020; Junrong et al., 2021). Among these powerful strategies, the transition metal-catalyzed C–H bond activation has long dominated the top topic in this field (Cho et al., 2011; Kuhl et al., 2012; Chen et al., 2015; Gensch et al., 2016). However, in the view of green and sustainable chemistry, (1) the utilization and removal of expensive transition metals like Rh and Pd, (2) the addition of extra oxidizing agents and additives, (3) the limitation of substrate scope and large-scale capacity, and (4) the relatively poor atom economy in removing guiding groups coordinated to the transition metal have further restrained its applications nowadays. Therefore, the development of novel strategies to address these aforementioned challenges in the functionalization of inert C–H bonds, especially the common and omnipresent C(sp^3^)–H bonds, is increasingly significant.

**FIGURE 1:**
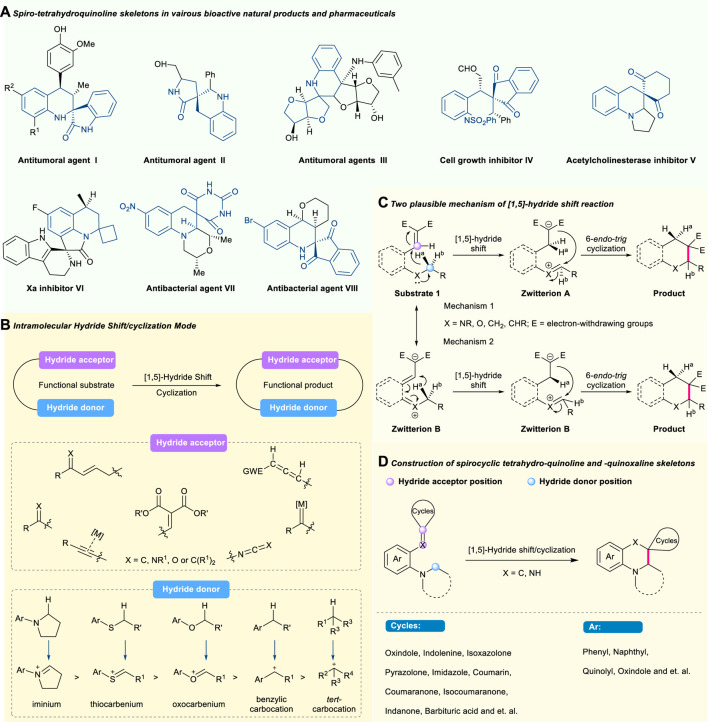
Selected bioactive natural products and pharmaceuticals with spiro-tetrahydroquinoline skeletons **(A)**, advancement of intramolecular **(B)** and two plausible mechanisms **(C)** of [1,5]-hydride shift mode, and construction of spiro-tetrahydroquinoline framework **(D)**.

With the continuing motivation towards green chemistry, the hydride shift-involved C(sp^3^)–H activation *via* a redox-neutral process, known as an ancient but effective methodology, provides new solutions to address those synthetic challenges (Wang and Xiao, 2016). In 1895, the phenomenon of redox-neutral C–H functionalization was first observed and then termed “*tert*-amino effect” by Meth-Cohn and Suschitzky in 1972 (Meth-Cohn and Suschitzky, 1972; Pinnow, 1895). Recognizing its great potential of selective activation and direct functionalization of unreactive C(sp^3^)-H bonds, enormous attention from the chemists has been paid to this magic effect, especially the most common migration mode of intramolecular [1,5]-hydride shift (Haibach and Seidel, 2014; Wang and Xiao, 2014; Kwon and Kim, 2016). Basically, both hydride donors and hydride acceptors are required in the hydride shift process (Figure 1B). The type of hydride donor involves C(sp^3^)–H bonds adjacent to *tert*-amines, ethereal oxygen and sulfur, benzylic C(sp^3^)–H bonds, and non-benzylic C(sp^3^)–H bonds, while the type of hydride acceptor contains electro-deficient alkenes, aldehydes, ketones, enals, enones, imines, alkynes, and allene derivatives. As for the specific mechanism of the hydride shift process, the scientific community has not been able to reach a consensus due to the two possible routes (Figure 1C). On the one hand (mechanism 1), the hydride shift process could undergo an intramolecular 6-*endo*-*trig* cyclization (or nucleophilic attack) to deliver the heterocycle after the generation of zwitterion A, which is formed by a [1,5]-suprafacial hydrogen shift from the α-position of carbon adjacent to heteroatom of substrate 1 to the electrophilic hydrogen acceptor in the form of a hydride (Nijhuis et al., 1987; Nijhuis et al., 1989; Meth-Cohn, 1996). On the other hand (mechanism 2), this transformation could be conducted through two sequential zwitterion B (the resonance form of substrate **1**) and A (as a result of the sequent [1,5]-suprafacial hydrogen shift in the form of a sigmatropic hydride shift from zwitterion B) to provide the target product (Datta et al., 2005; Odedra et al., 2007; Shu et al., 2008). To date, a series of critical reviews from reputable groups have summarized such great progress (Haibach and Seidel, 2014; Wang and Xiao, 2014; Kwon and Kim, 2016; An and Xiao, 2021). These reviews focus mainly on the application of hydride shift-involved C(sp^3^)–H activation to construct five-, six-, seven-, or other-membered hetero, spiro, or fused cycles, as well as acyclic multifunctional compounds. Among them, the intramolecular cascade [1,5]-hydride shift/cyclization sequence, as the most common and useful sequential reaction, is highly effective for C(sp^3^)–H bond activation/C–C and C–Heteroatom formation, and proves to be a versatile method to construct six-membered cyclic compounds, especially including spirocyclic molecules like spiro-tetrahydroquinolines (Rios, 2012; Mao et al., 2013; Wang et al., 2013; Zhu et al., 2017; Xu et al., 2019).

The spiro-tetrahydroquinoline skeleton as a privileged motif widely exists in biologically active natural products and pharmaceuticals (Figure 1A). For example, antitumoral agent (I), known as a novel synthetic molecule, exhibited antitumoral and antiplasmodial activities (Kouznetsov et al., 2010). Antitumoral agent (II) possessed good bioactivity to inhibit against the HeLa and MCF-7 cell lines at micromolar concentrations (Damerla et al., 2012). Antitumoral agent (III) showed obvious *in vitro* immunocompetence and cytotoxicity against Hela and Eca-109 cells (Liu et al., 2005). As a synthetic compound, cell growth inhibitor (IV) displayed significant wound-healing activities (Liou et al., 2021). Compound (V) was known as a potent inhibitor against acetylcholinesterase (Toth et al., 2018). Xa inhibitor (VI) demonstrated promising inhibitory activity in the micromolar concentration against serine protease (Medvedeva et al., 2018). Antitumoral agent (VII) showed potent activity against methicillin-resistant *Staphylococcus aureus* (MRSA) and fluoroquinoline-resistant bacterial strains (Ruble et al., 2009). The synthetic antibacterial agent (VIII) exhibited good antibacterial activity against microorganisms (Ramesh et al., 2009). During the past years, impressive advancements have been achieved in the synthesis of these molecules through organic or metal synthesis (Han et al., 2012; Shi et al., 2013; Wang et al., 2013; Yang and Du, 2013; Li and Du, 2014; Dong et al., 2021). However, severe challenges, like the utilization and removal of expensive transition metal, addition of extra oxidizing agents and additives, and poor atom economy, still exist in this field. Therefore, the development of a novel strategy to construct these valuable spirocyclic frameworks through direct functionalization of inert chemical groups or bonds is still highly demanded. As one of the most common and effective C(sp^3^)-H functionalization methodologies, the cascade [1,5]-hydride shift/cyclization strategy possessed an inherent advantage in the transformation of unreactive functional groups or bonds to various chemical structures. However, building structurally complex spiro-tetrahydroquinoline *via* the cascade [1,5]-hydride shift/cyclization strategy has rarely been established before the limited but significant works (Pastine and Sames, 2005; Kang et al., 2010; Cao et al., 2011; Wang, 2013; Mori et al., 2015; Zhu et al., 2017; Lv et al., 2019). More importantly, rarely an updated review has systematically summarized these important progresses despite scattered reports documented in several reviews (Wang and Xiao, 2014; Wang and Xiao, 2016; Xiao Mingyan et al., 2018; An and Xiao, 2021).

In this review, we will summarize the significant advances in the cascade [1,5]-hydride shift/intramolecular C(sp^3^)-H functionalization from the perspective of *tert*-amino effect to build a spiro-tetrahydroquinoline skeleton due to its great potential in the discovery of new drugs, and also point out its current limitations as well as future directions in this field. This review is categorized by the structure type of final spirocyclic products as follows (Figure 1D): Construction of a spiro-tetrahydroquinoline skeleton containing (1) (oxo)indole, (2) (iso)coumaranone, (3) pyrazolone and imidazole, (4) isoxazolone, (5) coumarin, and (6) other units.

## The Construction of a Spiro-Tetrahydroquinoline Skeleton Containing Various Pharmaceutical Cores

The spiro-tetrahydroquinoline skeleton as a privileged structure motif is quite frequently seen in a range of biologically active natural products and pharmaceuticals. In the process of constructing a spirocyclic tetrahydroquinoline framework *via* the [1,5]-hydride shift/cyclization strategy, the cyclic or acyclic *tert*-amino moiety of substrates always serves as a hydride donor, while electro-deficient alkenes containing various pharmaceutical cores (such as oxindole, indolenine, pyrazolone, coumarin, indanone, and coumaranone/isocoumaranone) and reactive imines function as a hydride acceptor, which fulfill the structural diversity of spirocyclic tetrahydroquinoline derivatives.

### The Construction of a Spiro-Tetrahydroquinoline or Tetrahydroquinoxaline Skeleton Containing an (oxo)Indole Unit

**SCHEME 1:**
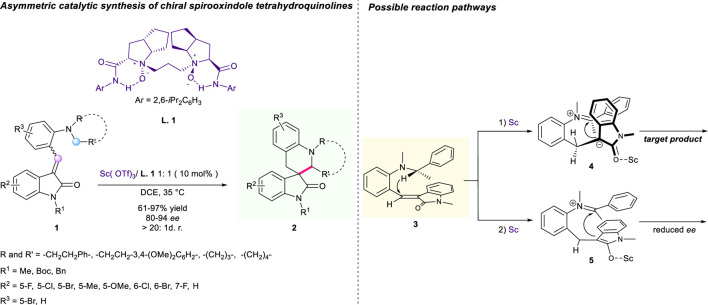
Asymmetric catalytic synthesis of chiral spirocyclic tetrahydroquinolines.

In 2015, Feng’s group developed an asymmetric tandem [1,5]-hydride shift/cyclization reaction to produce chiral spirooxindole tetrahydroquinolines using chiral *N*, *N*′-dioxide/Sc(OTf)_3_ as a catalytic system (Scheme 1) (Cao et al., 2015). As far as we know, this is the only report for the asymmetric version of tandem [1,5]-hydride shift/cyclization. The oxindole derivative **1** as a suitable hydride acceptor triggered the intramolecular tandem [1,5]-hydride shift/ring closure reaction. With the assistance of the chiral complex of *N*, *N*′-dioxide/Sc(OTf)_3_, all reactions proceeded smoothly in dichloroethane (DCE) at 35°C, offering a wide range of optically active spirooxindole tetrahydroquinolines **2** with high yields of up to 97% and excellent stereoselectivies of up to 94% ee and >20:1 dr. Moreover, a gram-scale investigation of this strategy was smoothly operated with excellent reaction performance, which demonstrated the robustness of this tandem sequence toward spirocyclic tetrahydroquinolines. The author proposed a possible mechanism of chiral memory effect dominating a helical chirality in a cationic intermediate to explain the chiral information observed in optically active products.

**SCHEME 2:**
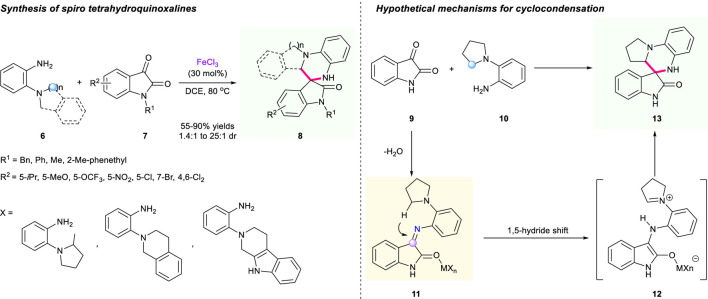
Construction of spiro tetrahydroquinoxalines *via* hydride shift/cyclization sequence.

In 2017, Tunge’s group revealed a Lewis acid-catalyzed synthesis of spiro tetrahydroquinoxalines from diamines **6** and isatins **7** (Scheme 2) (Ramakumar et al., 2017). The condensation of diamine **6** with the α-dicarbonyl substrate generated an imine intermediate **11** that is responsible for the [1,5]-hydride shift to nitrogen. Using FeCl_3_ as a promoter, various substituents at different positions of both substrates were all tolerated, yielding corresponding spirocyclic compounds X with accepted to outstanding yields (55%–90%) and moderate to excellent diastereoselectivities (1.4:1 to 25:1). A hypothetical mechanism for the key step of cyclocondensation is depicted in Scheme 2.

**SCHEME 3:**
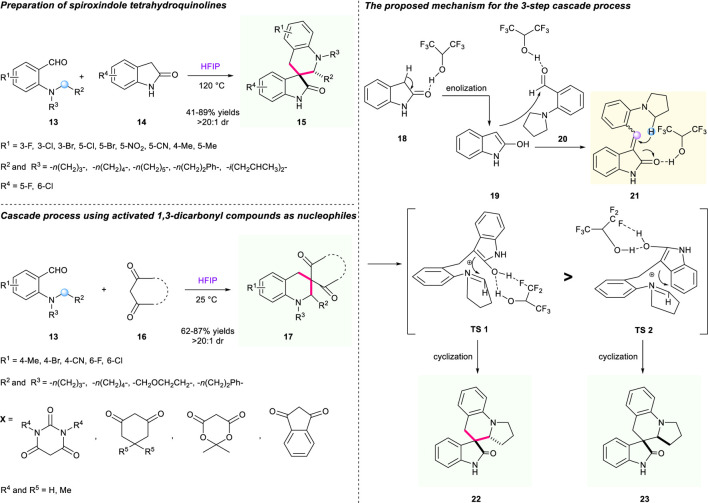
Efficient construction of tetrahydroquinolines *via* HFIP-mediated cascade [1,5]-hydride shift/cyclization.

One year later, Li’s group uncovered a fluorinated alcohol-mediated cascade [1,5]-hydride shift/cyclization reaction to prepare spiro-tetrahydroquinolines bearing oxindole moiety (Scheme 3) (Chen et al., 2018). Research indicated that hexafluoroisopropanol (HFIP) demonstrated a significant influence on the efficacy of the transformation. The Knoevenagel condensation of 2-(pyrrolidin-1-yl)benzaldehyde **13** and indolin-2-one **14** triggered a [1,5]-hydride shift/cyclization sequence to generate structurally diverse spirooxindole-fused tetrahydroquinolines. With the help of HFIP as a solvent, this strategy showed good tolerance of a variety of substrates, resulting in the corresponding products having moderate to good yields (32%–89% yield) with good to high diastereoselectivities (61:39 to >20:1 dr). A plausible mechanism displaying dual hydrogen bonds of HFIP with the enol moiety of intermediate **TS1/2** was proposed in their study, which is depicted in Scheme 3.

**SCHEME 4:**
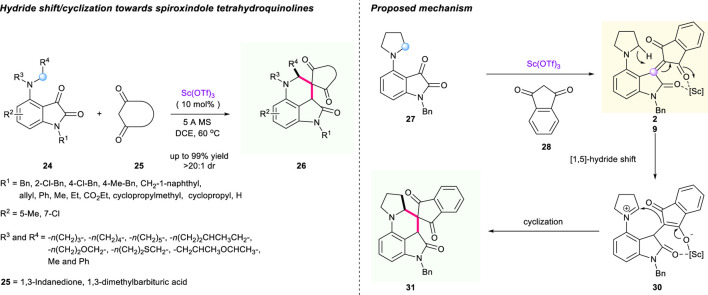
Cascade [1,5]-hydride shift/cyclization for synthesis of oxindole-fused tetrahydroquinolines.

Soon after, Xiao’s group uncovered the scandium-catalyzed redox-neutral cascade [1,5]-hydride shift/cyclization of C4-amine-substituted isatins **24** and 1,3-dicarbonyl compounds **25** (Scheme 4) (Zhu et al., 2019). In this process, the α,β-unsaturated 1,3-dicarbonyl intermediate **29** acted as a hydride acceptor. The optimized condition proved to be using dichloroethane (DCE) as solvent, 5-Å molecular sieves as additive, and Sc(OTf)_3_ as catalyst, delivering diverse product **26** with acceptable to good yields (48%–99% yield) and acceptable to excellent diastereoselectivities (1:1 to >20:1 dr). Intriguingly, the substrates containing asymmetrical acyclic *N*-benzyl-*N*-methylamine were also tolerated in this reaction, which has never been achieved before in most hydride shift sequences. Besides, the chiral control of the reaction was also investigated using chiral phosphoric acid as catalyst; however, only poor enantioselectivity was observed in this process. The plausible reaction mechanism is described in Scheme 4.

**SCHEME 5:**
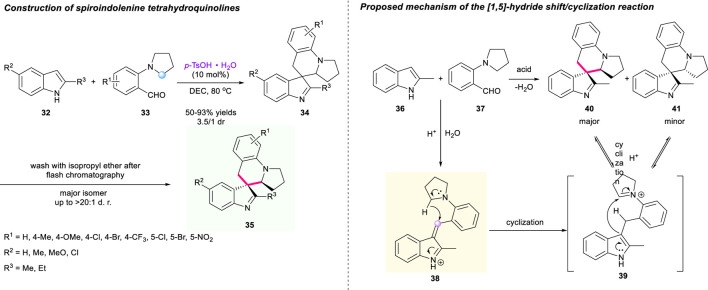
Concise approach to spiroindolenine tetrahydroquinolines.

Instead of the oxindole unit, indole substrates could also participate in the [1,5]-hydride shift/cyclization sequence. In 2015, Sun and Xu’s group revealed a concise approach to construct a spiro-tetrahydroquinolines incorporated indolenine moiety **34** *via* the [1,5]-hydride shift/cyclization sequence (Scheme 5) (Wang P.-F. et al., 2015). The hydride acceptor iminium **38**, formed through dehydration of substrates, induced the hydride shift/cyclization process under acidic conditions. Employing 2-substituted indoles **32** and 2-(pyrrolidin-1-yl)benzaldehydes **33** as substrates, *p*-TsOH·H_2_O as catalyst, and DCE as solvent, a wide range of desired target products **34** were successfully obtained with good to excellent yields and moderate diastereoselectivities. Interestingly, when the inseparable mixture of diastereoisomers was washed with isopropyl ether after rapid chromatography, the isolated products **35** could be obtained in up to >20:1 dr. An asymmetric version utilizing chiral BINOL-derived phosphoric acid was also conducted under the same condition, but only delivering the corresponding compound with low enantioselectivity. A plausible mechanism of this methodology is proposed in Scheme 5.

**SCHEME 6:**
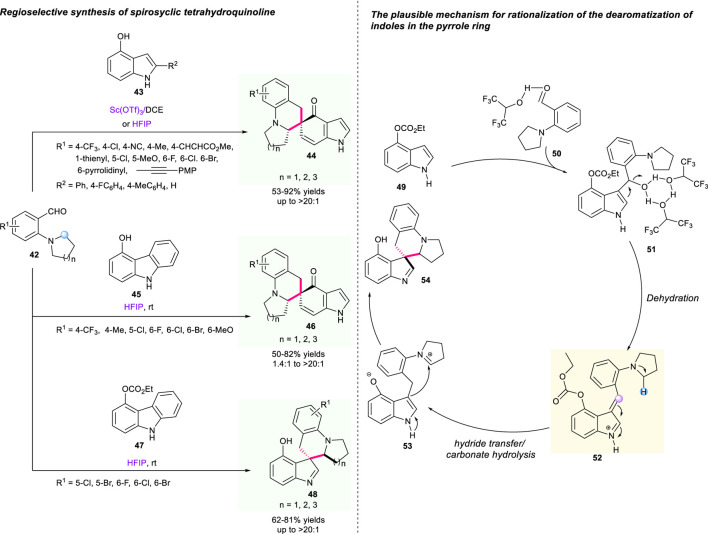
The regioselective dearomatization/hydride shift sequence to build spiro-tetrahydroquinolines.

Recently, Xiao’s group reported the first regioselective dearomatization between 4-hydroxindoles **43** or 4-hydroxycarbazole **45** and 2-aminobenzaldehydes **42** to construct spiro-tetrahydroquinolines *via* an aromatization-driven hydride shift strategy (Scheme 6) (Duan et al., 2020b). Under the catalysis of scandium complex and HFIP, a variety of spirocyclic molecules incorporating indoles and carbazole moieties were provided with moderate to high yields, respectively. Meanwhile, the author found that the protection of the OH group of 4-hydroxyindole with formic ester was preferred to the generation of the spiroindolenine in HFIP. To further explore the switchable dearomatization of indoles in the carbocyclic ring and pyrrole ring, a variety of 2-aminobenzaldehydes **42** reacting with ethyl (1H-indol-4-yl) carbonate **47** were examined, giving the corresponding spiroindolenines **48** a 62%–81% yield with up to >20:1 dr. A plausible mechanism indicated that the protection of the hydroxyl group of **47** shifted the direction to another reaction site and guaranteed the conduction of this reaction at the electron-rich C-3 position of indole. Undoubtedly, this strategy provided an answer to the limitation of switchable dearomatization of fused bicyclic aromatic compounds.

**SCHEME 7:**
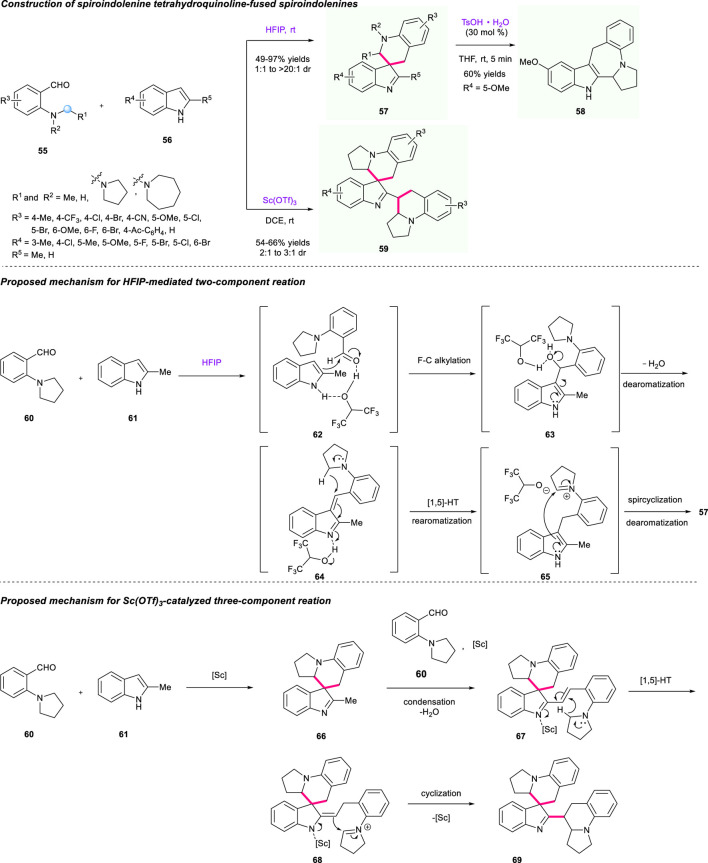
Divergent synthesis of tetrahydroquinoline-fused spiroindolenines.

The divergent synthesis of tetrahydroquinoline-fused spiroindolenines through cascade dearomatization of indoles with *ortho*-aminobenzaldehydes driven by the dearomatization force was also achieved by the same group (Scheme 7) (Shen et al., 2019). The type of substrate and catalyst was a critical factor in the regulation of divergent synthesis of the final spirocyclic products. Under the catalysis of HFIP acting as both solvent and reaction promoter, an array of tetrahydroquinoline-fused spiroindolenines **57** that contain diverse electron properties on the aromatical ring of both substrates **55** and **56** were efficiently synthesized at a 47%–97% yield with up to >20:1 dr. Moreover, the addition of TsOH·H_2_O could further enable the transformation of THQ-fused spiroindolenine to ring-expanded derivatives **58** *via* the 1,2-migration process. When adding Sc(OTf)_3_ as catalyst into the reaction instead of HFIP in DCE at room temperature, the three-component reactions for the assembly of tetrahydroquinoline-fused indolenines **59** were successfully achieved, giving the corresponding product a 54%–66% yield with 2:1 to 3:1 dr. The plausible mechanisms were described as shown in Scheme 7 to explain this divergent synthesis. The process of synthesizing products **57** mainly contained a Friedel-Crafts alkylation/hydrolyzation/[1,5]-hydride shift/spirocyclization sequence, which was similar to a previous work by Xiao’s group with the assistance of HFIP. As for product **69**, due to its strong Lewis acidity, Sc(OTf)_3_ was beneficial for generating the α,β-unsaturated indolenine intermediate **67** and then initiated [1,5]-hydride transfer/cyclization processes to provide the target products **69**.

**SCHEME 8:**
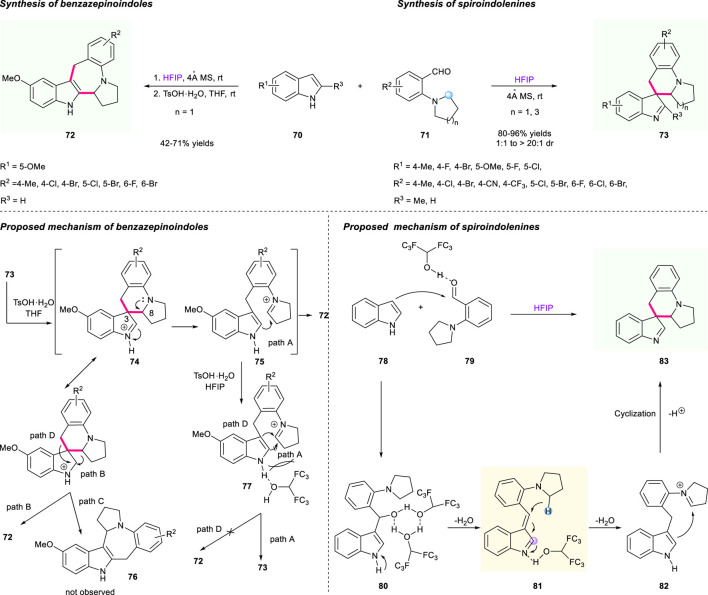
Controllable syntheses of spiroindolenines and benzazepinoindoles.

In addition to Xiao’s elegant work above, a controllable synthesis of spiroindolenines and benzazepinoindoles *via* HFIP-mediated cascade [1,5]-hydride shift/cyclization was successfully developed by the team of Li and Wang (Scheme 8) (Bai et al., 2019). As shown in Scheme 8 (top line), the controllable process of the two privileged skeletons **72**/**73** features high efficiency, mild reaction conditions, and good substrate tolerance, giving these two individual products a moderate to high yield (with moderate to excellent diastereoselectivities for product **73**). The proposed mechanism of rationalizing the formation of two products **72**/**73** was illustrated, and the addition of TsOH·H_2_O played an important role in this controllable process (Scheme 8, bottom line). As for the mechanism of spiroindolenines (Scheme 8, right column), the reactive intermediate **80** was formed by the promotion of HFIP-mediated H-bonding interaction between compounds **78** and **79**, followed by the result of vinylogous imine **81**, which was generated through dual hydrogen bond-promoted dehydration and served as a hydride acceptor. Under the activation of HFIP, the electrophilic iminium intermediate **82** was obtained *via* a [1,5]-hydride shift process, and then the dearomatization product **83** was furnished after the nucleophilic attack at the C3 of the indole moiety and cyclization sequence. As for the mechanism of benzazepinoindoles, the protonation of spiroindolenine **73** gave the intermediate **74**, which generated an iminium intermediate **75** after the bond cleavage of C3–C8 promoted by rearomatization. Then, the final thermodynamic benzazepinoindole **72** was offered after an attack of iminium moiety on the C2 position of the indole ring (path A). The author also proposed two alternative competitive migration processes based on the observed phenomenon (pathways B and C), which might furnish two possible products **72** and **76** *via* the “three-center-two-electron” transition state. However, in fact, there was no possible product **76** observed in this reaction. Notably, the N−H bond in intermediate **77** could chelate the OH group of HFIP with the addition of TsOH·H_2_O in the reaction medium, which served as a significant steric hindrance to block the nucleophilic attack of C2 of indole to the iminium moiety, and only delivered spiroindolenines **73** rather than benzazepinoindoles **72** (pathway D).

### The Construction of a Spiro-Tetrahydroquinoline Skeleton Containing an (iso)Coumaranone Unit

**SCHEME 9:**
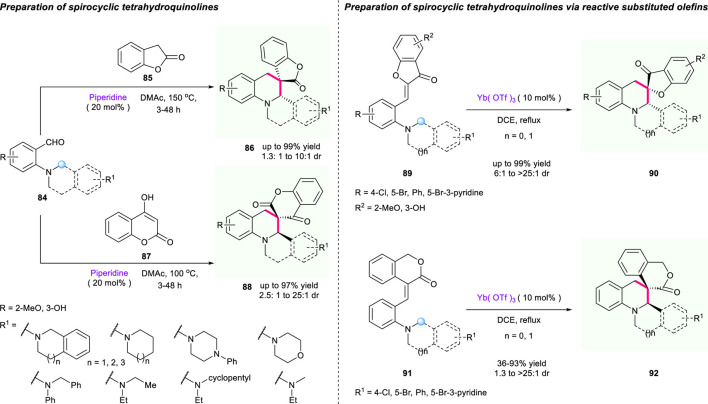
Cascade [1,5]-hydride shift/cyclization for synthesis of spirotetrahydroquinolines.

In 2020, Deb’s group reported a diastereoselective olefination/[1,5]-hydride shift/cyclization sequence to synthesize spiroheterocycles from reaction of *ortho* amino benzaldehydes **84** or olefins **85**/**87** with active methylene compounds **86**/**88** (Scheme 9) (Bhowmik et al., 2021). The α,β-unsaturated electron-deficient alkene used as a hydride acceptor enabled the synthesis of novel spiro tetrahydroquinolines bearing 2- or 3-coumaranone moieties with good to excellent yields (up to 99% yield). Moreover, the employment of 4-hydroxycoumarin or 3-isochromanone substituted olefins as substrates in the presence of Yb(OTf)_3_ successfully provided access to a wide range of spiro-tetrahydroquinolines **90**/**92** containing chromanone or 3-isochromanone moieties with excellent to good yields and diastereoselectivities.

**SCHEME 10:**
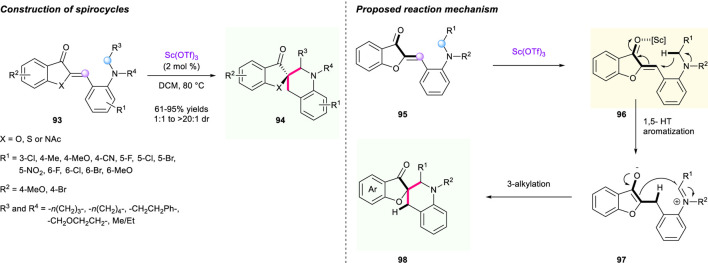
Facial access to [5.4] spirocycles *via* hydride shift reaction.

Soon after, an efficient access to tetrahydroquinoline spiro-heterocycles *via* the hydride shift cyclizations of aurones **94** was developed by Xiao’s group (Scheme 10) (Duan et al., 2020a). With low loading of Sc(OTf)_3_ in 2 mol%, a series of biologically important spiro-heterocycles were achieved with good yield (up to 95%) and good diastereoselectivities (up to >20:1 dr) under mild conditions. The researchers proposed a plausible mechanism for this reaction, as described in Scheme 10. Again, this work shows great potential of the driving force of aromatization in hydride shift cyclization strategy.

### The Construction of a Spiro-Tetrahydroquinoline Skeleton Containing Pyrazolone and Imidazole Units

**SCHEME 11:**
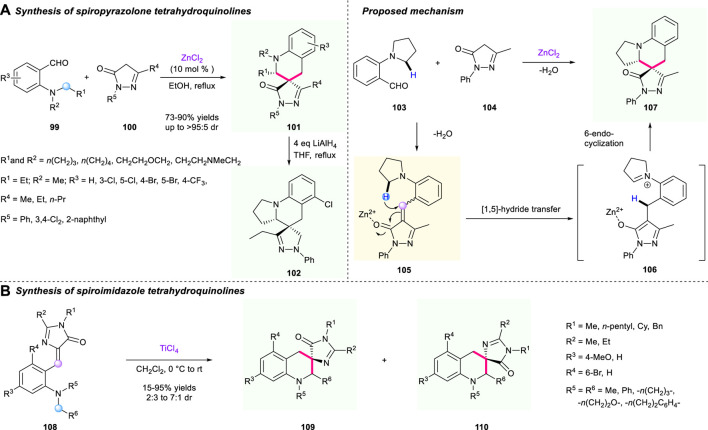
Concise synthesis of spiro-pyrazolone **(A)** and -imidazole **(B)** tetrahydroquinoline.

The structure of pyrazolone derivatives is widely seen in pharmaceuticals and drugs; hence, merging this valuable unit into a spiro-tetrahydroquinoline skeleton by a [1,5]-hydride shift/cyclization strategy could be a promising direction for the discovery of new drugs. In 2015, a zinc chloride-catalyzed protocol to synthesize a range of pyrazolone-fused spiro-terahydroquinolines *via* a tandem [1,5]-hydride shift/cyclization process was documented by Wang’s group (Scheme 11A) (Zhao et al., 2015). The α,β-unsaturated pyrazolone intermediate **101** served as a hydride acceptor and engaged in the 1,5-hydride shift/cyclization sequence. This methodology features broad substrate scope, high yields (up to 95% yield), good to excellent diastereoselectivities (up to >95:5 dr), as well as gram-scale capacity. Also, the reduction of one of the spirocyclic compounds **101** using LiAlH_4_ in refluxing THF condition was successfully realized, resulting in the corresponding novel spiro-terahydroquinoline **102** having a good reaction performance. However, efforts to explore an enantioselective version of this reaction are still being developed. The proposed mechanism for the construction of spiro-tetrahydroquinoline is shown in Scheme 11A.

Very recently, Smirnov’s group reported an intramolecular tandem [1,5]-hydride shift and cyclization to form spirocyclic tetrahydroquinoline derivatives **107** under the promotion of TiCl_4_ (Scheme 11B) (Zaitseva et al., 2021). The hydride shift process was triggered by reactive α,β-unsaturated imidazole fragments **105**. This reaction demonstrated impressive substrate tolerance, giving the desirable spirocyclic compounds a 25%–95% yield under mild conditions. Moreover, a gram-scale reaction was successfully conducted with up to 93% yield, paving the way to potential research of antibacterial activity of those bioactive molecules.

### The Construction of a Spiro-Tetrahydroquinoline Skeleton Containing an Isoxazolone Unit

**SCHEME 12:**
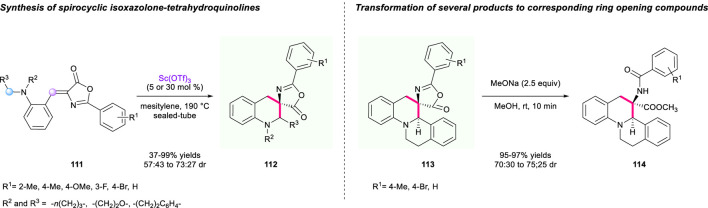
A tandem 1,5-hydride transfer/cyclization process to construct isoxazolone-tetrahydroquinolines and 3-amino-3-carboxytetrahydroquinoline derivatives.

As important heterocyclic structures, isoxazol-5-one and tetrahydroquinoline scaffolds are found in a wide range of medicines and bioactive natural products (Sridharan et al., 2011). Hence, merging these two structures to synthesize novel spirocyclic molecules could be attractive for the discovery of lead compounds. In 2013, an intramolecular tandem 1,5-hydride transfer/cyclization process catalyzed by Lewis acid Sc(OTf)_3_ to construct isoxazolone-tetrahydroquinolines and 3-amino-3-carboxytetrahydroquinoline derivatives has been established by the group of Yuan (Scheme 12) (Han et al., 2013). In this method, the (Z)-alkylidene azlactone **111** served as both a hydride donor and an acceptor, offering an array of tetracyclic and pentacyclic heterocycles containing two stereogenic centers and spirocyclic skeletons with up to 99% yield with diastereoselectivities ranging from 57:43 to 73:27. To demonstrate the synthetic utility of this method, transformation of several spirocyclic products to 3-amino-3-carboxytetrahydroquinoline derivatives **113** was also demonstrated through an efficient ring opening process using MeONa as base in MeOH with up to 97% yield, and 70:30 to 75:25 dr.

**SCHEME 13:**
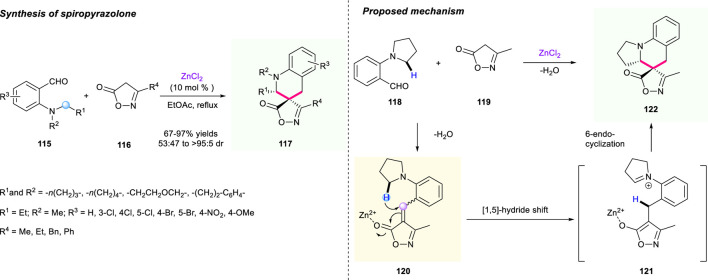
Synthesis of novel spiroisoxazol-5-one tetrahydroquinolines.

Three years later, Wang’s group designed a ZnCl_2_-tatalyzed Knoevenagel condensation/[1,5]-hydride shift/cyclization sequence to synthesize a series of novel spiroisoxazol-5-one tetrahydroquinolines (Scheme 13) (Zhao et al., 2016). In their strategy, the condensation of 2-(pyrrolidin-1-yl)benzaldehyde **118** and 3-methylisoxazol-5(4H)-one **119** changed the reactive intermediate **120** into a hydride donor and acceptor, which underwent a subsequent [1,5]-hydride shift/cyclization process to furnish the final product **122**. As a result, this reaction featured a broad substrate scope and a simple reaction operation, providing the target spirocyclic products **117** with up to 97% yield and up to >95:5 dr, which demonstrated the high efficiency of this methodology.

### The Construction of a Spiro-Tetrahydroquinoline Skeleton Containing a Coumarin Unit

**SCHEME 14:**
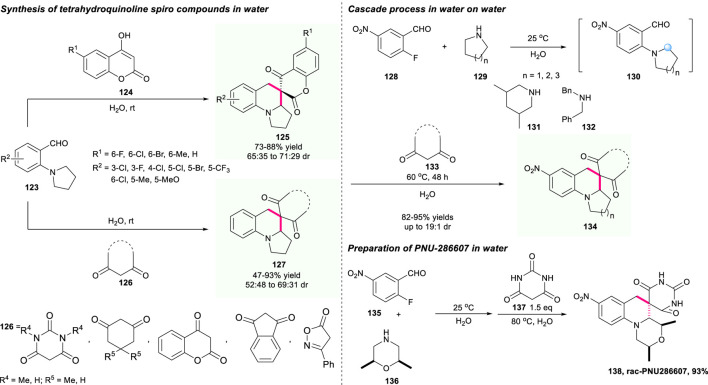
The water-promotion cascade reaction to synthesize spiro tetrahydroquinolines.

In 2017, Xiao’s group developed an innovative and operationally practical on-water catalysis to efficiently construct important spiro-tetrahydroquinoline compounds through a novel cascade S_N_Ar/Knoevenagel condensation/[1,5]-hydride shift/cyclization sequence (Scheme 14) (Zhu et al., 2017). Compared with previous work, this reaction offered an example of cascade C(sp^3^)–H functionalization sequence operated on water under mild conditions instead of complex and harsh reaction conditions. The Knoevenagel condensation of 2-aminobenzaldehydes **123** and 1,3-dicarbonyl **124**/**126** compounds generated the reactive electron-deficient alkenes, which further initialized the subsequent [1,5]-hydride shift/cyclization route. Most of the substrates could be well-tolerated and produce the required spirocyclic compounds **128**/**129** with good yield and acceptable dr values as well as good atom and step economy in one operation. Moreover, the construction of the anti-bacterial agent (−)-PNU-286607 was also smoothly conducted with an excellent yield of 93%, which further demonstrated the power of this strategy.

**SCHEME 15:**
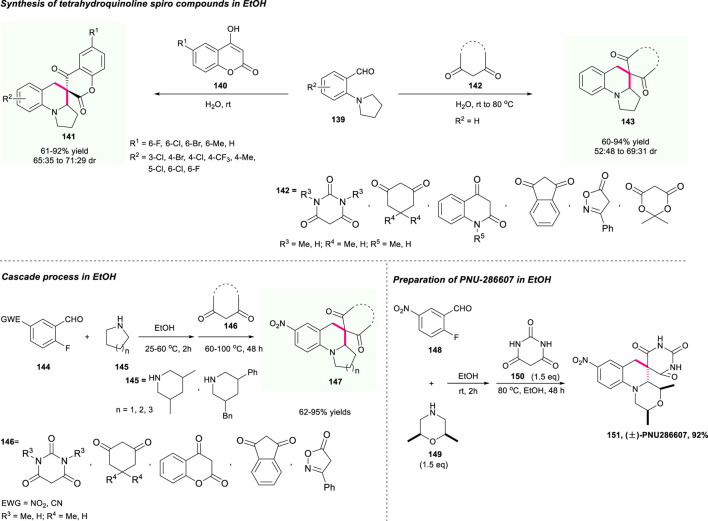
Facile synthesis of spirocyclic tetrahydroquinolines.

To further expand the potential of that practical strategy, in 2021, the same group developed a similar work that used environmental-friendly EtOH as solvent for the efficient construction of the pharmaceutically significant spirocyclic tetrahydroquinolines **147** (Scheme 15) (Yu et al., 2022). This strategy featured high efficiency, mild reaction conditions, high step and atom economy, and good substrate tolerance as well, producing the target spirocyclic tetrahydroquinolines containing different pharmaceutically interesting moieties with impressive results. Moreover, this strategy has been smoothly applied for the preparation of PUN-286607, affording the target molecule **151** with up to 92% yield, which demonstrated the powerful applicability of this method.

**SCHEME 16:**
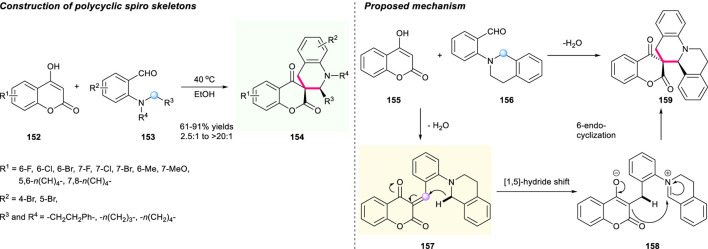
[1,5]-Hydride shift reaction toward spirocyclic tetrahydroquinolines bearing chromanone moieties.

In 2020, Wang’s group developed a catalyst-free tandem 1,5-hydride shift/cyclization process to form polycyclic spiro skeletons (Scheme 16) (Liu et al., 2020). The generated α,β-unsaturated chroman intermediate **157** acted as a hydride acceptor in the reaction process. This reaction features high atom and step economy, and mild conditions, providing access to a series of new spiro benzoquinolizidine-chromanones **154** with satisfactory yields (up to 91% yield) and excellent diastereoselectivities (up to >20:1 dr). Notably, both the gram-scale reaction and derivatization of the spirocyclic products were smoothly conducted with satisfactory reaction performance, which demonstrated the robustness of this methodology. A plausible mechanistic pathway was proposed by Wang and co-workers in Scheme 16.

### The Construction of a Spiro-Tetrahydroquinoline Skeleton Containing Other Units

**SCHEME 17:**
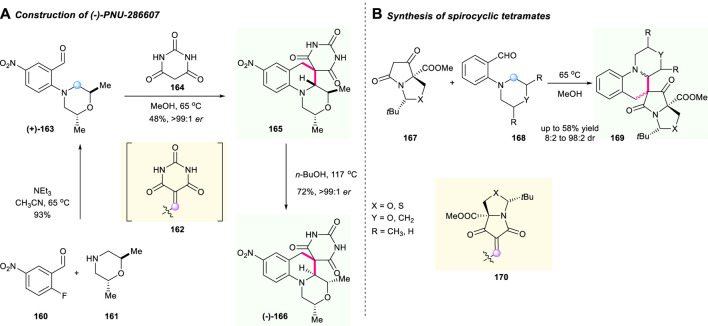
Asymmetric synthesis of (-)-PNU-286607 **(A)** and spirocyclic tetramates **(B)**.

As early as 2009, the Kamilar research group developed a practical two-step route for the asymmetric synthesis of the (-)-PNU-286607 **166**, a promising spirocyclic tetrahydroquinoline compound bearing barbituric acid moiety (Scheme 17A) (Ruble et al., 2009). This is a limited case that applied the cascade [1,5]-hydride shift/cyclization sequence to prepare the chiral barbituric acid-fused spiro tetrahydroquinoline. The whole reaction route started with chiral trans-dimethylmorpholine **161** in MeCN as a reaction mediated at a temperature of 65°C, and resulted in desirable chiral molecule **163** with excellent stereochemical control after a comprehensive study on the stereochemical process. Notably, the isomerization of **166** using *n*-BuOH as solvent delivered the diastereoisomer **165** with excellent reaction performance and excellent *ee* value. The condensation of aldehyde **160** and barbituric acid **164** led to the production of unsaturated barbituric acid intermediate **162** as a hydride acceptor. This exploration demonstrated the potential of [1,5]-hydride shift/cyclization-involved C(sp^3^)–H activation to construct valuable spiro-tetrahydroquinoline molecules.

Inspired by Kamilar’s unprecedented work, a sequential Knoevenagel condensation/[1,5]-prototropic shift of tetramates **167** with aminobenzaldehydes **168** to furnish the functionalized spirocyclic tetramates **170** was reported by Moloney’s group in 2019 (Scheme 17B) (Josa-Cullere et al., 2019). The α,β-unsaturated 1,3-dicarbonyl intermediate **169** served as a hydride acceptor and started the sequential reaction under optimized conditions. Interestingly, the stability of isolated major products was dependent on the solvent and on the nature of the azacycle, and the final products were obtained with low to good yields and up to 98:2 dr.

**SCHEME 18:**
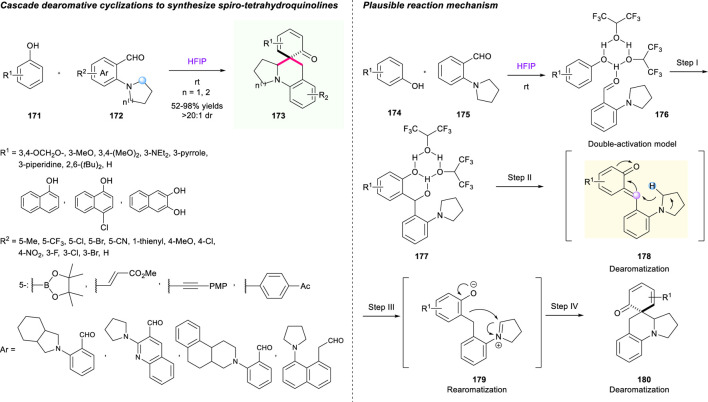
Synthesis of spirocyclic skeleton *via* cascade dearomative cyclization.

One year later, Xiao’s group developed a rapid buildup of polycyclic skeleton directly from phenols **171** and *ortho*-aminobenzaldehydes **172** *via* cascade [1,5]-hydride shift/dearomative cyclizations (Scheme 18) (Li et al., 2018a). HFIP was used as both reaction promoter and solvent, enabling one-step construction of structurally diverse spiro-tetrahydroquinolines with a good yield (up to 98%) and with high diastereoselectivities (up to >20:1), good functional group compatibilities, as well as gram-scale capacity. Importantly, this is an unprecedented strategy that employed *in situ* generated *o*-QMs **173** as novel hydride acceptors and aromatization as the driving force to initiate the hydride shift/cyclization sequence. Undoubtedly, this novel method opens a new avenue for the assembly of complex molecules *via* a cascade hydride shift/cyclization strategy. The researchers proposed a possible mechanism to point out the significance of HFIP (Scheme 18).

**SCHEME 19:**
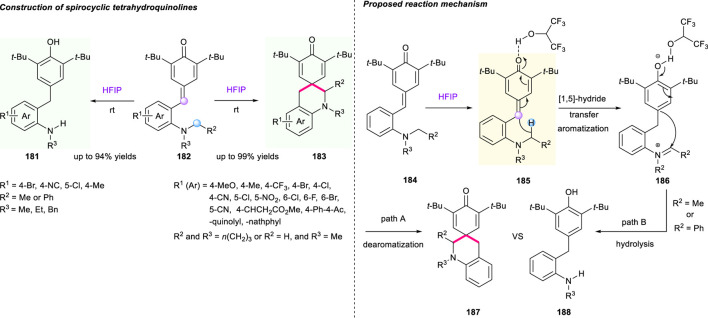
Aromatization-driven cascade sequence to prepare spirocycles.

In 2019, based on previous works, the same group continued to use HFIP as the solvent to develop *p*-QMs-triggered cascade [1,5]-hydride shift/spirocyclization and hydrolysis reaction. This strategy enabled the synthesis of spirocyclic products **181** with good to high yields (52%–99%) under mild conditions, featuring room temperature, additive-free, and good functional group tolerance (Scheme 19) (Lv et al., 2019). Interestingly, an array of *ortho*-benzylated anilines **183** were obtained with high yields when using acyclic amines incorporating *N,N*′-dibenzyl, *N*-methyl-*N*′-benzyl, and *N,N*′-diethyl groups. The plausible reaction mechanism indicated that the rearomatic complex **185** with iminium ion generated by the intramolecular [1,5]-hydride transfer underwent two reaction process to produce the dearomatic product **187** (path A) and the hydrolysis process product **188** (path B, *R* ^2^ = Me or Ph) based on the properties of *R* ^2^ groups. Undoubtedly, Xiao’s work demonstrated that aromatization serving as a powerful driving force could trigger hydride shift-involved cascade reactions for the buildup of architecturally complex molecules.

**SCHEME 20:**
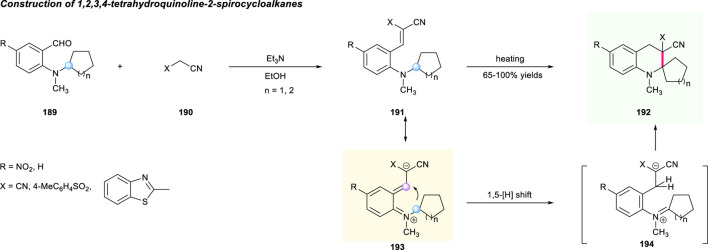
The construction of tetrahydroquinoline-2-spirocycloalkanes.

In addition to the above works, the [1,5]-hydride shift strategy can also be used for the construction of spirocyclic tetrahydroquinolines containing cycloalkane units. In 2006, Tverdokhlebov and co-authors disclosed an interaction of *ortho*-aminobenzaldehydes **189** with substituted acetonitriles **190** promoted by Et_3_N in EtOH to yield tetrahydroquinoline-2-spirocycloalkanes **192** with high yields (Scheme 20) (Tverdokhlebov et al., 2006). According to the *tert*-amino effect mechanism, the reaction was assumed to move forward *via* sequential Knoevenagel condensation/[1,5]-hydrogen shift/ring closure of the formed adduct.

## Summary and Prospect

Spiro-tetrahydroquinolines are unique molecules in medicinal chemistry and pharmaceuticals that have attracted considerable attention from the industrial and academic community. In the past years, remarkable advancements have been achieved in the construction of these useful compounds *via* the cascade [1,5]-hydride shift-involved C(sp^3^)–H activation reaction. In this review, we have systematically highlighted the utility and versatility of the cascade [1,5]-hydride shift/cyclization reaction for constructing spiro-tetrahydroquinoline derivatives. These valuable spirocyclic molecules have been well categorized according to the structural type of final products. Despite the significant developments that have been made in this growing field, some challenges still need to addressed: (1) The limitation of substrate scope, structural diversity of product, complex reaction conditions, and problem of large-scale capacity still limit its potential in organic synthesis (Mori et al., 2014; Liu et al., 2018; Xing et al., 2020; Yuan et al., 2020; Guo et al., 2021; Sakai et al., 2021; Yang X. et al., 2021; Xie et al., 2022). (2) The current application of the cascade [1,5]-hydride shift/cyclization strategy mainly focuses on the construction of five- and six-membered spiro-tetrahydroquinoline. However, exploration of building a spiro-architecture with a challenging ring size (like divergent synthesis of medium ring size) as well as conducting total synthesis of a structurally complex natural product remain elusive (Li et al., 2018b; Wang et al., 2018; Kataoka et al., 2019; Hu et al., 2020; Shen et al., 2020; Hu et al., 2021a; Hu et al., 2021b; Wang et al., 2021; Yang S. et al., 2021). (3) Notably, the application of [1,5]-hydride shift/cyclization strategy in stereoselective chemistry was barely reported (Mori et al., 2018). Considerable efforts should be focused on the synthesis of chiral spirocyclic molecules with the application of this powerful strategy. (4) Finally, the bio-evaluation of target spirocyclic products for new drug discovery and research is quite far behind its synthetic chemistry (Sridharan et al., 2011; Wang Y. et al., 2015; Muthukrishnan et al., 2019). Further medicinal research of those bioactive compounds should be devoted to this field in the near future. We hope our review could provide a quick look into and offer some inspiration for the research on hydride shift strategy in the future.
